# 4DCT is long overdue for improvement

**DOI:** 10.1002/acm2.13933

**Published:** 2023-03-03

**Authors:** Erik Tryggestad, Heng Li, Yi Rong

**Affiliations:** ^1^ Department of Radiation Oncology Mayo Clinic Rochester Minnesota USA; ^2^ Department of Radiation Oncology John Hopkins University Baltimore Maryland USA; ^3^ Department of Radiation Oncology Mayo Clinic Phoenix Arizona USA

**Keywords:** four‐dimensional computed tomography, motion management, radiotherapy

## 1 INTRODUCTION

Technology innovation has always been one of the main drivers for the evolution of the radiation oncology field. New commercial devices are emerging at a fascinating speed, that is, Magnetic Resonance Imaging (MRI)‐based linac, Positron Emission Tomography (PET)‐based linac, FLASH ultra‐high dose rate delivery, ring gantry with double stacked multi‐leaf collimators (MLC), etc. Meanwhile, technology that has already been widely adopted and used for many patients seems to have stagnated. 4DCT was first introduced in 2003, exactly 20 years ago,^1^ aiming to reconstruct “dynamic volumetric imaging” or “respiration‐correlated CT”. It adds a temporal dimension to the traditional 3DCT and provides visualization of target motion. Subsequent clinical trials for anatomy sites pertaining to moving targets imposed a vastly reduced planning target margin using 4DCT.^2^, ^3^ The entire field has benefited from the addition of this technology, with informed knowledge of its limitations, that is, motion uncertainties and image artifacts resulted from the irregularity of patients' breathing patterns. From a clinical physicist's point of view, 20 years have gone by, and the developments on 4DCT seem to be mostly on paper, with minimum to none being incubated to mature commercial products. We cannot help but wonder *should we call for vendors' attention to devote resources in innovating 4DCT technology since it is long overdue for commercial developments*, or *should we be made aware that incremental developments have been trialed out, but fundamental limitations of 4DCT are hard to be overcome*? We are now passing these questions to two experienced physicists with expertise in motion management and 4DCT. Dr. Erik Tryggestad argues for the proposition that “4DCT is long overdue for improvement” while Dr. Heng Li argues against it.

Dr. Erik Tryggestad is an Associate Professor and Consultant of Medical Physics in the Department of Radiation Oncology at Mayo Clinic, Rochester (MN), having recently marked 10 years working at Mayo's Proton Beam Therapy Center. Prior to his faculty positions at Mayo, he was an Assistant Professor at Johns Hopkins University in the Department of Radiation Oncology. Dr. Tryggestad earned his PhD in experimental nuclear physics from Michigan State University's National Superconducting Cyclotron Laboratory in 2001 and ultimately transitioned to medical physics as a post‐doctoral research fellow at the end of 2004 at Johns Hopkins University. Dr. Tryggestad works in the clinic covering proton therapy and Gamma Knife radiosurgical procedures. His research and translational interests include motion management, image guidance, and aspects of radiotherapy workflow automation (especially AI‐based autosegmentation).  Dr. Tryggestad has contributed to over 75 peer‐reviewed publications in scientific journals.

Dr. Heng Li is an Associate Professor in the Department of Radiation Oncology and Molecular Radiation Sciences at Johns Hopkins University and is a fellow of the American Association of Physicists in Medicine. He serves as the Chief Proton Physicist of the Johns Hopkins Proton Therapy Center, located at the Sibley Memorial Hospital, Washington DC. He completed his PhD in Electrical and Computer Engineering at the University of Virginia, Charlottesville, VA in 2006, and received postdoctoral fellowship and therapeutic medical physics residency training at the University of Texas MD Anderson Cancer Center. His research interests include proton therapy physics, motion management in radiotherapy, global health, and AI application in radiation oncology. He has published over 70 scientific papers in peer‐reviewed academic journals and authored 9 book chapters.

## 2 OPENING STATEMENT

### 2.1 For the proposition: Erik Tryggestad, PhD

I entered the field at the tail end of 2004, taken under the wing as a post‐doctoral fellow by one of our most impactful image‐guided radiotherapy (IGRT) innovators, Dr. John W. Wong (FAAPM, Quimby Awardee), just as 4DCT was being translated clinically. Synergistically (no pun intended; thanks partly to JWW and his colleagues), the year before, the first cone‐beam CT (CBCT)‐guided linear accelerators were introduced.^4^, ^5^ On‐board kV 2D, 2D fluoroscopy, 2D/3D, kV CBCT, and MVCT imaging catapulted us to the modern 4D‐IGRT era.

The first methods for 4DCT were described in 2003 for helical or conventional CT and CBCT (e.g.,^1^, ^6^). Consequently, 4DCT‐informed “motion management” based on 4D‐CT simulation became a hot topic.^7^ Indeed, for the first time we had a 4D radiotherapy treatment planning (RTP) tool providing a patient‐specific, 3D target‐motion estimation. Thus, the Internal Target Volume (ITV)‐based approach for RTP leveraged this more explicit ITV, significantly reducing either a population‐based 3D margin expansion or an envelope based on multiple conventional/3D CT scans.^8^, ^9^ Almost overnight, 4DCT revolutionized RTP for gated RT deliveries,^10^, ^11^ allowing for hypothetical reduction in this patient‐specific ITV corresponding to a portion of respiration. Meanwhile, thanks to the near‐elimination of inter‐fraction setup margins^12^ afforded by on‐board 3D IGRT, new 4DCT informed vigor was injected into the concept of “probabilistic” RTP (e.g.,^13^, ^14^, ^15^, ^16^, ^17^, ^18^, ^19^, ^20^, ^21^, ^22^, ^23^). On paper, it was shown that 4D‐probabilistic plans could reduce prescription treatment volumes such that they could compete with gating and tumor tracking while using the full respiratory duty cycle, yielding an optimal efficiency.^16^, ^19^, ^22^, ^23^ Along the way, deformable image registration (DIR)^24^, ^25^ filled a big gap as the enabling technology for the more generic emergence of “4D RTP,” be that for 4D dose accumulation (retrospective evaluation) or implicit with 4D optimization (which we might now refer to as robust 4D planning). More recently, pencil beam scanning (PBS) for protons and ion beams has pushed the envelope in this respect given heightened concerns over motion interplay compared with conventional x‐rays.^26^ Commercial offerings for robust 4D planning now exist and 4D dose accumulation considering PBS delivery timing information is recommended as “vital” for PBS clinical deployment by the AAPM Task Group 290.^27^

Here's my frustration: ***Twenty years*** since initial adoption of 4DCT on conventional CT scanners and with the hindsight of all the 4DCT‐enabled innovations that followed, if we evaluate how 4DCT simulation is predominantly deployed today, we find that 4DCT's original limitations clearly persist. True, we've gone from ∽16‐ to ∽128‐slice CT simulators, making 4DCT acquisition slightly faster. But, 4DCT is dose constrained; therefore, it forcibly represents a snapshot in time. Clinical 4DCT is predominantly built upon retrospective sorting (purely from a 1D external surrogate) using slice stacking. Binning is predominantly based purely on *either* phase or amplitude (with preference towards the former given the probabilistic advantages).

***News flash***: *RT patients do not breathe like programmable robots* providing the smooth and predictable respiration that most of our 4D validation phantom and in‐silico studies are based upon. Because our patients do not breathe like robots, and because 4DCT imaging has not significantly evolved, ***we are often forced to work clinically with a 4DCT simulation image that is artifact‐prone in the context of variable breathing*** (i.e., containing volume inconsistencies). Granted, our predominant 4DCT deployment using *only* a 1D external motion surrogate information simplifies downstream IGRT. But, physicists, how do we clinically cope with an artifact‐ridden 4DCT simulation image? How can we anticipate those patients for which we've failed to capture a truly representative snapshot of breathing and accurate motion baseline? At my esteemed institution, we're essentially generating 4DCT with the same surrogate device and method described originally in,^6^ despite the fact that many in academia have been working hard on incremental improvements to 4DCT over these 20 years (no offense to my friend and former colleague, Dr. Eric Ford).

One excellent incremental improvement example was published by Olsen et al., wherein the authors proposed a hybrid amplitude‐percentile binning strategy to reduce volume inconsistencies.^28^ This simple technique does not require fancy image processing and results in equivalency with the probabilistic (equal‐time weighted, phase) bins we've come to rely upon. I even hijacked this method for my earliest foray into 4D‐MRI^29^ and it helped deal with our basic real‐world problem of breathing irregularity. I tell this story because I think it calls attention to commercial 4DCT stagnation and long‐term disconnect that seemingly persists.

For all the good things enabled by improvements to robust 4D planning infrastructure, let's work on *mainstream translation* of “4DCT 2.0!” Better sorting schemes, potentially involving internal image surrogates, have been shown to reduce volume inconsistencies.^28^, ^30^, ^31^ Despite its well‐understood flaws, DIR can help.^32^, ^33^, ^34^ Artificial intelligence (AI) can help with noise reduction of lower‐dose projections enabling oversampling.^35^, ^36^ Roughly 15 years of academic investigation has demonstrated that MRI can generate a representative 4D image leveraging longer acquisition times (e.g.,^20^, ^21^, ^37^, ^38^), although, historically, MRI is fraught with prohibitive signal‐to‐noise ratio issues in the thorax. Perhaps 4DCT and 4D‐MRI can join forces to overcome their respective deficiencies?

### 2.2 Against the proposition: Heng Li, PhD

4DCT is the standard tool for simulation and treatment planning for radiation therapy of mobile tumors. Taking the proposition literally, “4DCT is long overdue for improvements” implies that no improvements, or not many, have been made for a long time. However, since the early 2000s, when 4DCT was introduced,^39^, ^40^ there has been a massive body of literature on the topic. I will review some of the literature below that showed (1) the tremendous improvement of 4DCT over the past two decades and (2) the fundamental problems of 4DCT that incremental improvements could not address.

#### 2.2.1 Simulation: Image quality and artifacts

4DCT is used to visualize the respiratory motion during simulation. The patient's respiratory motion leads to inconsistencies in data for conventional free breathing 3DCT (fan‐beam CT, FBCT) reconstruction. The 4DCT technique reduces/removes the inconsistency by binning the CT data into different breathing phases. It is important to note that 4DCT techniques assume regular breathing patterns so that the data can be correctly assigned to individual phases. As such, breathing irregularities could cause motion artifacts and inaccuracies in quantifying target motion.^41^ Indeed, one study suggested that 4DCT image artifacts correlated with worse local control in SBRT of lung and liver metastases.^42^ Tremendous efforts have been made, which has resulted in a better understanding of contributing factors and mitigation strategies to reduce 4DCT motion artifacts^43^, ^44^ and enhance image qualities.^45^, ^46^ However, because of the relatively short 4DCT acquisition time, it is difficult to acquire sufficient data for artifact‐free 4DCT reconstruction with the presence of irregular breathing. Alternative techniques, including 5D CT^32^ and 4D MRI,^41^ which both allow much longer acquisition time, mitigation strategies to reduce the degree of breathing irregularities,^41^ or independent review of 4DCT scans,^47^ are possible solutions to the problem—but either are outside the scope of improving 4DCT or have not reached maturity for commercialization.

#### 2.2.2 Treatment planning: Target delineation

4DCT is used for target delineation in the radiotherapy of mobile tumors. The process often involves delineating target volume on individual phases, the maximum intensity projection (MIP), or the average intensity projection data sets. It has a clear advantage over FBCT‐based target delineation, which assumes the target is static. Still, the accuracy of the 4DCT‐based delineation is also the subject of acquisition techniques and could be impacted by image artifacts. In 2011 and 2018, two multi‐institute 4DCT phantom studies revealed similar results where 4DCT‐based delineation could lead to significant volume deviations, whereas the amplitude deviation was generally small but could be systematic.^48^, ^49^ Certainly, more accurate target delineation would lead to a reduced need for margins and improved treatment plan quality. In recognizing the need to quantify target delineation uncertainties, a more recent study showed that a comprehensive QA of 4DCT could quantify the system's performance. The authors proposed acceptable volume and amplitude deviation criteria after fine‐tuning acquisition parameters and evaluating results from various phantom studies, with regular and irregular breathing patterns.^50^

#### 2.2.3 Treatment delivery

4DCT‐based treatment planning achieved superior results compared to conventional FBCT‐based plans, even when the FBCT‐based plan employed generous margins.^51^ Whereas, framing 4DCT as an integrated part of radiation treatment, one could argue that 4DCT merely captures a snapshot of the patient's respiratory motion at simulation. After considering the irregular patient‐specific respiratory motion, the delivered dose to the patient could deviate from the 4DCT‐based dose calculation.^52^ Therefore, improving dose calculation accuracy by incorporating patient breathing patterns during treatment is undoubtedly desirable. However, understanding or capturing a full picture of a patient's breathing pattern, instead of a snapshot, might not be realistically achievable, especially for patients with difficulty breathing or other health issues. It is likely that, any incremental improvement of 4DCT, might theoretically mitigate the uncertainty issue or 4DCT artifacts, but clinically might not make any significant differences. Instead, investigation on real‐time volumetric imaging at the treatment position, for example, MR‐linac, or active motion management techniques such as breath hold or gating, are more likely to improve the accuracy of delivered dose calculation.

In summary, 4DCT has undergone continuous and substantial improvement over the past two decades since its invention, yet the same fundamental issues persist. In other words, it is unrealistic to expect any incremental improvement with clinical significance in 4DCT as long as its limitation is tied to unsolvable issues, that is, inconsistency and irregularity in the patient's breathing pattern.

## 3 REBUTTAL

### 3.1 For the proposition: Erik Tryggestad, PhD

Well played, Dr. Li! Paraphrasing from my esteemed friend's summary arguments: 4DCT has a fatal flaw which is revealed in the context of irregular breathing; incremental improvements will not overcome this. I agree with Dr. Li that we should have grounded and realistic expectations. However, we must ask: has mainstream (i.e., clinically adopted) 4DCT reached its technological ceiling? Most emphatically, “no it has not!”

Irregular breathing is far from occasional in the radiation oncology setting.^20^, ^21^, ^53^, ^54^, ^55^, ^56^, ^57^ Therefore, 4DCT reconstruction artifacts stemming from this problem are both significant and extremely common.^33^, ^58^, ^59^ However, based on my own 18 years of clinical experience (most recently, 10 years spent in the proton therapy realm where we take great care to monitor 4DCT stability) most of the time we do tend to capture our patients' correct expiratory baseline (even for “irregular breathers”). As such, an achievable goal for 4DCT is to improve upon our definition or concept of “representative motion from the observed baseline.”

I believe that our innovative community has already identified the key elements that can and should comprise the mainstream concept of “4DCT 2.0.” We have an excellent benchmark, which both Dr. Li and I cited above, coming from Dr. Dan Low and colleagues, namely “5DCT.”^31^, ^32^, ^33^ 5DCT involves acquisition of N (e.g., 25) conventional (low‐dose) fast helical CTs along with the recording of a traditional 1D external surrogate; then 3D DIR (CT_1_‐CT_N_) is used, along with analysis of the respiratory surrogate, to reconstruct an average 3D CT representation for any user‐selected breathing phase or bin. Three elements of 5DCT are important, if not crucial: (1) a significantly oversampled CT acquisition, (2) sampling of given slices of anatomy evenly throughout the full acquisition duration to minimize systematic bias, and (3) use of a 1D respiratory signal that has an analog or correlate with the device to be used for downstream IGRT. The main gaps (naively, as I conceive of them) are technologies that enable significant dose reduction (commensurate with the needed oversampling boost) and companion decision‐enabling tools that allow the physicist to interact with and evaluate the reconstruction in a transparent fashion. Importantly, evaluation of the respiratory surrogate waveform in advance of, or even during, CT acquisition could (dynamically) inform the choice of oversampling rate.^60^ In practice, I believe the only concession we need to be willing to make is with respect to time: “4DCT 2.0” necessarily must take slightly longer to acquire.

### 3.2 Against the proposition: Heng Li, PhD

I agree with my opponent that the 4DCT's original limitations have persisted since its inception. That is, the inability to handle irregular breathing, and as a snapshot of the patient's respiratory motion, it does not necessarily represent the patient's breathing during treatment. However, we disagree if improvements in 4DCT technology could meaningfully mitigate these limitations.

Indeed, as both my opponent and I agree, numerous incremental improvements in the 4DCT technique have led to better images with fewer artifacts. However, what impact do these improvements have on radiotherapy simulation, treatment planning, and delivery?

Dr. Tryggestad suggested that improved binning in 4DCT could help remedy the irregular breathing problem. Indeed, it was shown that amplitude‐based (including hybrid) binning could reduce image artifacts compared to phase‐based binning. However, that may not necessarily translate into better imaging for radiotherapy. Li et al. compared target delineation results using amplitude‐based and phase‐based binning 4DCT images. They illustrated that while amplitude‐based binning reduced image artifacts, it also underestimated the full range of motion, partly because it does not use the full range of breathing data for reconstruction.^61^ The irony is that ITV delineation with (phase‐based) 4DCT has already been shown to result in large variation because of irregular breathing in a simulation study.^62^ In short, coupled with the relatively short acquisition time of 4DCT, irregular breathing of patients is not going to be resolved by incremental improvement of 4DCT, including different binning techniques. In principle, methods capable of long acquisition time, including 5DCT^32^ and 4DMRI,^41^ could help mitigate the problem. Patient training, coaching, and feedback have also been shown to be practical ways to remove/reduce the breathing irregularity.

Certainly, coaching or not, there is still concern that the 4DCT simulation may not represent the patient's breathing pattern during treatment. Persson et al.^63^ suggested that large interfraction variation is presented in breathing amplitude irrespective of audio coaching. They suggested that daily image guidance for verification of respiratory pattern and tumor motion should be performed, which I agree. It is probably outside of this debate to go into more detail about patient verification imaging and its relationship with 4DCT, other than pointing out that fundamentally, improving 4DCT will not impact its ability to predict how a patient is going to breathe during treatment.

All these being said, I believe there is room for 4DCT to improve, but there are limits and inherent uncertainties with 4DCT. Physicists must understand these limits and adequately handle the uncertainties associated with 4DCT.

## AUTHOR CONTRIBUTION

ET and HL contributed equally to the manuscript writing. YR contributed to topic conception and manuscript writing.

## CONFLICT OF INTEREST STATEMENT

The authors declare no conflicts of interest.
